# Acute generalized exanthematous pustulosis

**DOI:** 10.1186/1939-4551-8-S1-A194

**Published:** 2015-04-08

**Authors:** Luana Pires Helal, Liziane Nunes, Paula Lauria, Ana Luiza Cotta De Alencar Araripe, Elisa Fontenelle Oliveira, Sandra Bastos, Abelardo Neto, Celso Ungier, Flavia Anisio, Mariana Stoll Leão

**Affiliations:** 1 Instituto Nacional De Saude Da Mulher, Da Criança e Do Adolescente Fernandes Figueira - IFF/ Fiocruz, Brazil

## Background

Acute generalized exanthematous pustulosis (AGEP) is a rare and severe subtype of drug eruption, characterized by acute, extensive, non-folicular, sterile pustules overlying an erythematous skin, associated with fever and leukocytosis.

## Methods

Case report of a 6-year-old female child with encephalopathy and chronic lung disease, who was treated with intravenous antibiotics, antipyretic and antiepiletics during hospitalization for sepsis. She evolved with diffuse erythemata and non follicular pustules on her trunk, flexural areas, palms and soles.

## Results

The drug history, clinical presentation, cutaneous histopathological findings (subcorneal pustular dermatitis) and the prompt regression of the lesions after withdrawal of the suspected drugs were consistent to ratify the diagnosis of AGEP.

## Conclusions

AGEP is an uncommon reaction , usually seen after drug exposure and clinicians should keep in mind the possibility of this skin eruption.

## Consent

Written informed consent was obtained from the patient for publication of this abstract and any accompanying images. A copy of the written consent is available for review by the Editor of this journal.

